# Adenocarcinoma and polyposis of the colon in a 20-year-old patient with Trisomy 13: a case report

**DOI:** 10.1186/s13256-018-1600-8

**Published:** 2018-03-04

**Authors:** Danielle P. Thurtle, Michael B. Huck, Kristen A. Zeller, Tamison Jewett

**Affiliations:** 1 Wake Forest School of Medicine, Medical Center Blvd, Winston Salem, NC 27157 USA; 2 Lehigh Valley Health Network, 1200 S Cedar Crest Blvd, Allentown, PA 18103 USA

**Keywords:** Trisomy 13, Patau syndrome, Adenocarcinoma, Polyposis, Colorectal Cancer

## Abstract

**Background:**

Trisomy 13 is one of the most common autosomal trisomies, and although increasing in number, patients surviving past the neonatal period remain rare. The natural history and expected complications in these patients as they age remains unknown. Despite the rarity of this condition, unusual malignancies have been reported in the medical literature for decades. It is clear that providers should suspect unusual malignancies in these patients, particularly as they age.

**Case presentation:**

We report a 20-year-old Caucasian woman with Trisomy 13 who presented with colonic volvulus, found to have colonic polyposis and adenocarcinoma of the colon. Genetics of pathology specimens revealed 47(XX) + 13 without other mutations. She underwent prophylactic completion colectomy due to presumed risk of colorectal cancers given underlying adenomatous polyposis. She has recovered well without evidence of recurrence.

**Conclusions:**

The presence of colonic polyposis and colorectal cancer without family history or known mutations for polyposis syndrome suggests an intrinsic predisposition toward colorectal cancer in this patient with Trisomy 13. Recent research into colorectal cancer oncogenes supports that aneuploidy or increased copy number of certain genes on chromosome 13 may increase the risk of malignant transformation. This is an important correlation for researchers studying these topics and clinicians caring for patients with Trisomy 13 as they age.

## Background

Trisomy 13 is one of the most common autosomal aneuploidies, with a prevalence of 1.18–1.39 cases per 10,000 live births in the United States [1]. Mortality is very high for this condition, with a median survival of 2.5–10 days and less than 10% surviving to 1 year [2–5]. Given the rarity of children with Trisomy 13 surviving to adulthood, the clinical complications and expected pathologies in such patients as they age are not well known.

Increased incidence of malignancy in patients with constitutional Trisomy, including Trisomy 13, 18, and 21, has been described previously. Specifically for Trisomy 13, a recent review found 17 malignant tumors reported in the literature [6]. The most common reported malignancies include embryonic tumors, predominantly neuroblastoma and nephroblastoma, followed by malignant germ cell tumors, leukemias, brain cancer, carcinomas, and sarcomas. Two cases of carcinomas, an adrenal carcinoma and an adenocarcinoma of the colon, both noted incidentally on autopsy findings of neonates, have been reported [7, 8]. Although a small number of reports, this is exceptional given how rare carcinomas are in neonates. Despite this relatively increased incidence of unusual neoplasms in neonates with Trisomy 13, the mechanism responsible for development of malignancy in these patients is unknown. We describe one of the oldest known patients with Trisomy 13 reported in the literature, the second case of colorectal cancer in a patient with Trisomy 13, and the first case of colonic polyposis. The presence of colonic polyposis suggests a predisposition toward adenocarcinoma of the colon that is important not only in the clinical setting as more patients with Trisomy 13 age into adulthood but may also be pertinent to research concerning malignant transformation.

## Case presentation

We present the case of a 20-year-old Caucasian woman with Trisomy 13 (non-mosaic) with colonic polyposis and adenocarcinoma of the colon. She was originally diagnosed with Trisomy 13 as an infant via karyotype that was ordered due to multiple congenital anomalies. Her medical history includes ventriculoseptal defect that was closed as an infant, seizure disorder, profound developmental delays leaving her without purposeful speech and requiring gastrostomy dependence for all nutrition, spastic quadriplegic cerebral palsy, cataracts, and urinary tract anomalies including horseshoe kidney, and frequent urinary tract infections. At her medical baseline she is on numerous antiepileptic medications, urinary tract prophylaxis, and vitamins. She lives at home with her father with home health nursing support.

**Fig. 1:**
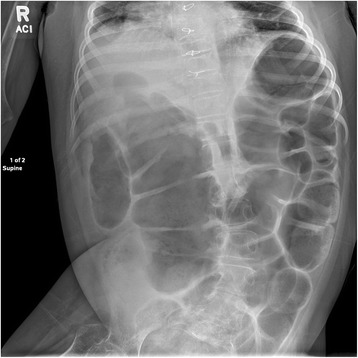
Extensive colonic dilatation without evidence of pneumatosis or pneumoperitoneum was identified on plain abdominal film at the time of admission and was similar to an Xx-ray obtained 1 week prior

She was brought to the hospital on day of admission by her home health nurse due to bloody output from her gastrostomy tube and abdominal pain. Notably, she was discharged from the hospital 2 days prior after brief admission for Pseuodmonas aeruginosa and *Escherichia coli* urinary tract infection causing increased seizure frequency. At that time, she was started on a 14-day course of ciprofloxacin, which she was still taking at home. Her vital signs on admission were normal and she was apyretic. A physical examination was significant for abdominal distention and tenderness to palpation, hypoactive bowel sounds, and a decreased level of alertness compared to her neurologic baseline. Laboratory findings were significant for a white blood cell count of 21,900, but other hematologic and electrolyte results were normal. Urinalysis was positive for trace protein, small leukocytes, but negative for nitrite. Point-of-care gastroccult and hemoccult testing were positive. An X-ray of her abdomen was significant for pancolonic gaseous distention with a loop of large bowel, which was noted to be similar to baseline abdominal imaging (Fig. 1). She was made non per oral, started on proton pump inhibitors, and previous ciprofloxacin and home medications were continued. Blood and urine cultures, Clostridium difficile, and other infectious testing for gastrointestinal pathogens returned negative results.

**Fig. 2:**
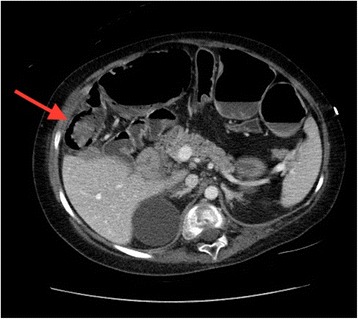
Pneumatosis of the right colon and significant colonic distention identified on computed tomography scan. The arrow is pointing to pneumatosis of the right colon

Twenty-four hours after admission, she developed an acute abdomen and signs of septic shock with white blood cell count increased to 26,300. Antibiotic therapy was broadened to intravenous metronidazole and vancomycin. Computed tomography of her abdomen with intravenous contrast revealed ascending ischemic colitis involving the cecum and ascending colon with pneumatosis and portal venous gas (Fig. 2).

She was taken to the operating room urgently and exploratory laparotomy revealed her cecum was not fixed in the right lower quadrant and the ascending and transverse colon had volvulized around the ileocolic pedicle. She had three areas of full-thickness necrosis in the ascending and transverse colon. The intervening segments of colon were viable. She had a normal ligament of Treitz. The left colon had proper fixation. Although there were no areas of perforation, there was murky ascites in the abdomen and pelvis. Because our patient was hypotensive requiring pressors during the case, the decision was made to perform a resection and bring out an ileostomy with creation of a Hartmann’s pouch. She completed a 10-day course of intravenous meropenem. She did well postoperatively, enteral nutrition was advanced slowly, and she was discharged home on postoperative day nine.

The pathology report of her resected colon revealed ischemic necrosis and ulceration, as well as an incidental finding of a 2-cm adenocarcinoma. The resection margin was clear with all 16 lymph nodes negative for disease, indicating a diagnosis of stage 1 cancer of the transverse colon (T2N0M0). Microarray and karyotype of the malignancy revealed 47XX + 13 without any other identifiable cytogenetic abnormality. Pathology also noted numerous other tubular adenomas concerning for polyposis of the remaining colon. There was no family history of familial polyposis syndromes or colorectal cancers. She subsequently underwent completion proctocolectomy due to polyposis and risk of developing additional cancers in the future.

Pathology of the proctocolectomy specimen confirmed polyposis with multiple tubular adenomas with high-grade dysplasia and benign lymph nodes. The postoperative course was complicated by small bowel obstruction due to an internal hernia around her ileostomy requiring surgical reduction, and portal vein thrombosis, which spontaneously resolved. Over 2 years after her completion proctocolectomy she is doing well without signs of local recurrence or metastasis.

## Discussion

The findings of colonic polyposis and adenocarcinoma of the colon in a young adult with no such family history and no known mutations associated with either condition suggests Trisomy 13 may be an important clinical factor. Chromosomal aneuploidy is thought to be an important step in malignant transformation of cells [9]. The exact risk that constitutional trisomy of chromosome 13 confers for malignancy is unknown. Trisomy 13 as the sole cytogenetic abnormality has been implicated in the malignant transformation of acute leukemias, especially myeloid leukemias, since the early 1990s [10, 11]. This has been described as a rare but distinctive lineage that confers poor prognosis [12]. Chromosomal analysis of adenomatous colonic polyps has shown trisomy of chromosome 13 to be a frequent abnormality, thought to contribute to malignant transformation [13–15]. The exact mechanism of this contribution remains unknown, but an increased copy number of certain genes on 13q have been implicated in colorectal carcinomas [16]. In particular, cyclin-dependent kinase 8 (*CDK8)* is a known colorectal oncogene, also located on 13q, and has been found to have increased copy number in 62% of colorectal cancers [17–20]. None of these genetic variants have been noted in patients with a polyposis syndrome, and it is unclear how Trisomy 13 without other cytogenetic abnormalities confers an increased risk of colonic polyposis.

Colonic adenomatous polyposis is a condition characterized by numerous adenomatous polyps. It is associated with heritable genetic syndromes, most frequently familial adenomatous polyposis (FAP) and MUTYH-associated polyposis [21]. Polyposis typically indicates an increased risk of colorectal cancer and is usually associated with an underlying premalignant genetic abnormality [22]. About 20% of colonic polyposis cases are considered sporadic, meaning patients do not test positive for known syndromic gene mutations [21]. A majority of these are still familial, with numerous family members exhibiting polyposis, indicating a likely genetic association as yet undescribed [21]. There is no known association between aneuploidy or increased copy number of genes on chromosome 13 and colonic polyposis.

## Conclusions

The finding of polyposis syndrome and colorectal cancer in an adult patient with Trisomy 13 without other cytogenetic abnormalities indicates a predisposition to developing colorectal cancer, but the molecular basis for this remains unclear. Given recent discoveries surrounding the importance of chromosome 13 on the malignant transformation of colorectal cancers, it is possible this is not a coincidence. This is an important correlation to report not only for researchers, but also for clinicians caring for patients with Trisomy 13. It is clear that clinicians should suspect unusual malignancies in these patients, particularly as they age.

## Authors’ information

Dr. Thurtle is a pediatric hospitalist and assistant professor in the department of pediatrics at Wake Forest School of Medicine in North Carolina. Dr. Huck is a fellow of colon and rectal surgery at the Lehigh Valley Health Network in Pennsylvania. Dr. Zeller is a pediatric surgeon and assistant professor at Wake Forest School of Medicine. Dr. Jewett is a medical geneticist and professor of pediatrics at Wake Forest School of Medicine.
